# Photocrosslinkable Nanofibrous Asymmetric Membrane Designed for Wound Dressing

**DOI:** 10.3390/polym11040653

**Published:** 2019-04-10

**Authors:** Patrícia Alves, Marta Santos, Sabrina Mendes, Sónia P. Miguel, Kevin D. de Sá, Cátia S. D. Cabral, Ilídio J. Correia, Paula Ferreira

**Affiliations:** 1 CIEPQPF, Department of Chemical Engineering, Universidade de Coimbra, P-3030 790 Coimbra, Portugal; martapatricia17@hotmail.com (M.S.); sabmendes@gmail.com (S.M.); icorreia@fcsaude.ubi.pt (I.J.C.); 2 CICS-UBI, Health Sciences Research Center, Universidade da Beira Interior, P-6200 506 Covilhã, Portugal; soniapmiguel@gmail.com (S.P.M.); ringo.kesa@gmail.com (K.D.d.S.); catiadcabral@gmail.com (C.S.D.C.)

**Keywords:** electrospinning, asymmetric membrane, skin regeneration, biocompatibility

## Abstract

Recently, the biomedical scientists who are working in the skin regeneration area have proposed asymmetric membranes as ideal wound dressings, since they are able to reproduce both layers of skin and improve the healing process as well as make it less painful. Herein, an electrospinning technique was used to produce new asymmetric membranes. The protective layer was composed of a blending solution between polycaprolactone and polylactic acid, whereas the underlying layer was comprised of methacrylated gelatin and chitosan. The chemical/physical properties, the in vitro hemo- and biocompatibility of the nanofibrous membranes were evaluated. The results obtained reveal that the produced membranes exhibited a wettability able to provide a moist environment at wound site. Moreover, the membranes’ hemocompatibility and fibroblast cell adhesion, spreading and proliferation at the surface of the membranes were also noticed in the in vitro assays. Such results highlight the suitability of these asymmetric membranes for wound dressing applications.

## 1. Introduction

Skin is the outer layer barrier of the body and hence it is frequently exposed to different hazard agents, which may lead to the occurrence of wounds [1]. A wound occurs when the normal anatomic structure and functions of skin are compromised. Surgical and accidental lacerations, burns, and pressure and diabetic ulcers are mainly responsible for the occurrence of skin injuries [2]. Nowadays, the treatment for this type of wounds has been attained essentially using autografts and allografts. However, these therapeutic approaches suffer from limited availability of healthy donor sites and immune rejection [3]. 

To overcome these shortcomings, myriad three-dimensional (3D) skin substitutes have been developed and are already available in the clinic [4]. These 3D skin constructs are classified as epidermal, dermal and epidermal/dermal substitutes depending on the degree of severity of the wound [5]. Among them, the epidermal/dermal substitutes (e.g., Apligraf^®^, Intergra^®^, and Orcel^®^) are the most innovative, since they have incorporated within their 3D matrix keratinocytes and fibroblasts, giving them the potential to regenerate both epidermal and dermal layers of skin. However, they have associated high production costs as well as some cases of immune rejection [6,7]. 

In this way, researchers have been involved in the development of wound dressings able to mimic the native features of skin and, simultaneously, make the wound healing process less painful and faster. Among the different dressings produced, those with an asymmetric geometry are the ones that more accurately reproduce the physical structure of the normal skin [8,9,10,11]. The main advantage of having an asymmetric wound dressing lies in addressing two distinct layers: (i) the top protective layer that acts as primary wound cover and prevent the penetration of pathogens and dehydration of the skin; and (ii) the underlying layer, which is in contact with the damaged skin, and is characterized by high porosity and hydrophilicity suitable to absorb the exudates, maintaining the hydrated environment essential for an effective skin regeneration [12,13].

Different techniques (e.g., wet-phase inversion, dry/wet phase inversion, supercritical fluid-phase inversion and electrospinning) can be used to produce asymmetric membranes [5,14]. Among them, the electrospinning technique presents several advantages over other methods used, since it allows the production of nanofibrous wound dressings with a structure very similar to that of the extracellular matrix (ECM) of the native skin [15]. Furthermore, the electrospun membranes exhibit a high surface area-to-volume ratio that promotes cell attachment and interconnected porosity favorable to gas exchange, nutrient supply and control of fluid loss [12,16]. In addition, the polymer electrospun membranes can themselves achieve hemostasis since their porous structure can facilitate quick absorption of blood and formation of pressure on the wound [17]. 

Therefore, this paper describes the production and characterization of an electrospun asymmetric membrane aimed for skin regeneration. The protective barrier of this membrane was produced using a blend of synthetic polymers (polycaprolactone (PCL) and polylactic acid (PLA)), while the underlying layer was composed of a mixture of natural polymers (chitosan (Ch) and gelatin (Gel)). The choice of these materials was based on previous studies showing electrospun scaffolds based on natural polymers successfully enhance tissue regeneration [18,19,20,21]. These phenomena are attributed to specific amino acid sequences present on the structure of proteins, such as gelatin, which promote cell-surface recognition as well as adhesion, spreading, activation, migration, proliferation and differentiation [22]. On the other hand, chitosan containing scaffolds present other benefits such as improvement of wound healing process due to its structural similarity to glycosaminoglycans in the natural tissues’ EMC [23]. In addition, chitosan is a polymer that possesses additional and significant properties that include its antifungal activity and bacteriostatic ability [24]. 

The polymers used in the production of bottom layer were previously modified with methacrylic anhydride. We previously reported the use of methacrylated gelatin (GelMA) in the production of electrospun PCL/GelMA meshes [18]. However, in this work, such functionalization methodology was adopted to modify both gelatin and chitosan to produce a bioactive blended bottom layer (GelMA/ChMA) to be incorporated in an asymmetric electrospun membrane.

Once modified, the polymers were mixed with the photoinitiator Irgacure^®^ 2959 and the final mixture was then electrospun and afterwards photocosslinked under UV irradiation. The suitability of the asymmetric electrospun membrane as wound dressing was evaluated through the determination of their chemical/physical/biological properties.

## 2. Experimental Section

### 2.1. Materials

3-(4,5-Dimethylthiazol-2-yl)-2,5-diphenyltetrazolium bromide (MTT) was purchased from Alfa Aesar (Ward Hill, Haverhill, MA, USA). Normal Human Dermal Fibroblasts (NHDF) cells were obtained from PromoCell (Labclinics, S.A., Barcelona, Spain). Fetal bovine serum (FBS) was acquired from Biochrom AG (Berlin, Germany). Gelatin type A (from porcine skin, 300 bloom), methacrylic anhydride (MAA), polycaprolactone (PCL, *M*_n_ ~80 kDa), poly(l,d-lactic acid) (PLA) IngeoTM2002D, with a reported average *M*_w_ of approximately 130,000 g/mol, were purchased from NatureWorks (Naarden, The Netherlands). Amphotericin B, Dulbecco’s modified Eagle’s medium (DMEM-F12), gentamicin, phosphate-buffered saline solution (PBS), chitosan (low mol wt 50,000–190,000 Da and 75–85% deacetylated) and trypsin were obtained from Sigma-Aldrich (Sintra, Portugal). Deuterium oxide was obtained from Euriso-Top, (Saint-Aubin, France). Glacial acetic acid (AAc), 2,2,2-trifluoroethanol (TFE), chloroform (CLO), dimethylformamide (DMF) and dimethyl sulfoxide (DMSO) were purchased from Fisher Scientific (Porto Salvo, Portugal). The photoinitiator 1-[4-(2-hydroxyethoxy)phenyl]-2-hydroxy-2-methyl-1-propan-1-one (Irgacure^®^2959) was provided by Ciba Specialty Chemicals (Basel, Switzerland). Anticoagulated rabbit blood (ACD blood) was purchased from PROBIOLÓGICA (Biologic Products Company) (Lisbon, Portugal) and used on the same day it was received.

### 2.2. Functionalization of the Biopolymers

The preparation of GelMA was performed as described elsewhere [25,26]. Briefly, a solution of gelatin 10% (*w*/*v*) in PBS pH 7.4 was prepared at 50 °C. Afterwards, 1% (*w*/*v*) of methacrylic anhydride was added under vigorous magnetic stirring. The mixture was left to react for 1 h at 50 °C and was then dialyzed against distilled water for 3 days and finally frozen and freeze-dried.

ChMA was also prepared through the reaction of low molecular weight chitosan with methacrylic anhydride. Initially, a 2.5 (*w*/*v*) solution of chitosan was prepared in a 2% (*v*/*v*) solution of acetic acid. Once solubilization was complete, 1% (*w*/*v*) of methacrylic anhydride was added to the solution under magnetic stirring. The mixture could react during 1 h at 50 °C and the obtained product was purified and freeze-dried as previously described for gelatin.

### 2.3. Electrospinning

Fibrous meshes were prepared using an electrospinning set-up composed of a high voltage power supplier (SL 10W-300W, Spellman, West Sussex, UK), a syringe pump (NE-1000 Multiphaser, New Era Pump Systems, Farmingdale, NY, USA) and a rectangular copper collector.

#### 2.3.1. Protective Barrier

The protective barrier was prepared by the blending between PCL and PLA polymers. For this, PCL and PLA solutions were prepared by dissolution of 1.5 g of each polymer in 10 mL of a CLO/DMF mixture (7:3, *v*/*v*). These solutions were then vigorously stirred until homogenization was attained. The final solution (PCL/PLA) was then electrospun using a flow rate of 2.5 mL/h, an applied voltage of 17 kV and using a working distance of 15–20 cm. 

#### 2.3.2. Underlying Layer

The underlying layer was composed of GelMA and ChMA solutions, which were prepared separately. GelMA was dissolved in 10 mL of a mixture of TFE/AAc (4:1, *v*/*v*) at a concentration of 10% (*w*/*v*). In turn, ChMA was dissolved in 10 mL of a 2% (*w*/*v*) AAc solution at a concentration of 3% (*w*/*v*). The polymeric solutions were then mixed in a 7:3 proportion of GelMA:ChMA until homogenization be achieved. Afterwards, 10 mg of the photoinitiator Irgacure^®^ 2959 was added to the polymeric solution. After that, the GelMA/ChMA solution was electrospun, using a flow rate of 1.3 mL/h and an applied voltage of 18.4 kV. The GelMA/ChMA fibers were collected over the previously prepared PCL/PLA fibrous mesh. Individual GelMA/ChMA fiber meshes were also prepared for ATR-FTIR, dynamic contact angle measurements and weight loss and cytotoxicity evaluation.

### 2.4. Photocrosslinking of the Fibrous Membranes

The crosslinking of the functionalized biopolymers (GelMA and ChMA) of the fibrous meshes was performed by UV. Before UV irradiation, samples of the fibrous membranes were placed in a closed container with a saturated solution of pentahydrated copper sulfate for a period of 24 h. After that, the fibrous membranes were exposed to UV light (Multiband UV UVGL-48 Mineral Light^®^ Lamp, wavelengths of 254–354 nm) for 10 min per side. Finally, the membranes were washed with distilled water and dried at 25 °C in a vacuum oven.

### 2.5. Characterization of the Fibrous Meshes

#### 2.5.1. Nuclear Magnetic Resonance (NMR)

The ^1^H NMR spectra of the biopolymers before and after grafting were obtained on a Bruker Avance III 400 MHz with a magnetic operation field at 9.4 Tesla spectrometer. Gelatin and GelMA samples were dissolved in deuterated water (D_2_O). Chitosan and ChMA were dissolved in D_2_O with 1% of acetic acid.

#### 2.5.2. Fourier Transform Infrared-Attenuated Total Reflectance Spectroscopy (FTIR-ATR)

The FTIR-ATR spectra were recorded using a JASCO FT-IR-4200 spectrometer equipped with a Golden Gate Single Reflection Diamond ATR accessory in the range of 500–4000 cm^−1^ at 64 scans with a resolution of 4 cm^−1^. 

#### 2.5.3. Scanning Electronic Microscopy (SEM)

The morphology of both the protective and underlying layers’ surfaces of the asymmetric membrane were analyzed through SEM analysis. The samples were placed on a double-sided graphite tape, attached onto a metal surface and sputter-coated with gold for 10 s. SEM images were acquired at 1 kV with a FE-SEM Zeiss Merlin Gemini 2 (Carl Zeiss, San Diego, CA, USA). The size of fiber diameters and their distribution were measured from SEM images, using the image analysis software ImageJ (ImageJ 1.46r; 2012, Bethesda, MD, USA).

#### 2.5.4. Dynamic Contact Angles Measurement

The water contact angles of the asymmetric membranes were assessed for both of their surfaces and for each fiber mesh produced individually. Contact angles were measured through a Dataphysics OCA-20 contact angle analyzer (Dataphysics Instruments, Filderstadt, Germany) using the sessile drop method. A droplet of deionized water (10 µL) was dispersed onto the sample surface and its evolution along time was recorded with a CCD video camera attached to the equipment. From the film frames, the water contact angles along time were automatically calculated by the equipment software.

#### 2.5.5. Weight Loss Evaluation

To evaluate the stability of the crosslinked samples in contact with PBS solution pH 7.4, the weight loss was determined. To accomplish that, the PCL/PLA and GelMA/ChMA fibrous mats before/after UV irradiation were weighed, placed in contact with PBS and incubated at 37 °C for 3 days. After that, the samples were rinsed with distilled water, and dried in a vacuum oven at room temperature. The weight loss after incubation was calculated using Equation (1).

(1) $$Weight\;loss\;\left(\%\right)=\frac{W_{i}-W_{f}}{W_{i}}$$

where W*_i_* is the sample’s initial weight and W*_f_* is the sample’s weight after incubation.

#### 2.5.6. Blood Compatibility

Blood compatibility assays were done in vitro according to the International Standard Organization (ISO) 10993-4 [27]. Thus, the hemolytic potential and thrombogenicity were determined for all produced membranes (n = 3). In either case, anticoagulated rabbit blood (ACD blood) was used during the assays.

##### Hemolysis

The hemolysis tests were accomplished as described in the American Society for Testing and Materials (ASTM) F 756-00 standard [28]. Briefly, asymmetric membranes displaying an area of 21 cm^2^ were incubated at 37 °C for 72 h in 7 mL of PBS (10 M, pH = 7.4). Then, the samples were removed and incubated with diluted anticoagulated rabbit blood (ACD blood) (10 ± 1 mg/mL) at 37 °C for 3 h, and gently inverted twice every 30 min, assuring the contact of the materials with blood. The positive (+) (total hemolysis) and negative (−) controls were achieved through the addition of rabbit blood in distilled water and PBS solution, respectively. After centrifugation at 750× g (15 min), the hemoglobin released by hemolysis ([Hb]) was measured by optical density of the supernatants at 540 nm using a spectrophotometer UV–Vis (Jasco V550). The percentage of hemolysis (HI) was determined according to Equation (2).

(2) $$HI=\frac{{\left[Hb\right]}_{test}-{\left[Hb\right]}_{negative\;control}}{{\left[Hb\right]}_{positive\;control}-{\left[Hb\right]}_{negative\;control}}\times 100$$

According to ASTM F 765-00 [28], materials are classified as non-hemolytic when 0 > HI > 2, slightly hemolytic when 2 > HI > 5 and hemolytic when HI > 5.

##### Thrombogenicity

The thrombus formation on the surface of both sides of the asymmetric membranes was evaluated following the gravimetric method reported by Imai and Nose (1972) [29]. Briefly, 250 µL of ACD blood were placed in contact with the materials surface. In the positive control, ACD blood were put in contact with an empty glass Petri dish. Blood clotting tests were initiated by adding 25 µL of a 0.1 M calcium chloride solution and were stopped after 40 min through the addition of 5 mL of distilled water. The resultant clots were fixed with 5 mL of a 1 mL of 36% (*w*/*w*) formaldehyde solution and dried at 37 °C, until constant weight. The percentage of thrombogenicity was determined by using Equation (3).

(3) $$\%thrombogenicity=\frac{m_{test}-\;m_{negative\;control}}{m_{positive\;control}-\;m_{negative\;control}}\times 100$$

### 2.6. Proliferation of Normal Human Dermal Fibroblasts in Contact with the Nanofibrous Membranes

NHDF cells were cultured in DMEM-F12, enriched with 10% heat inactivated FBS, amphotericin B (100 μg/mL) and gentamicin (100 μg/mL) in 75 cm^2^ culture T-flasks. Cells were maintained in a humidified atmosphere at 37 °C, with 5% CO_2_. Prior to cell seeding, each individual mesh was put in 96-well plates and sterilized by UV exposure for at least 1 h. Then, NHDF were seeded at a density of 10 × 10^3^ cells/well to evaluate the cell adhesion and proliferation in the presence of the membranes. Cell growth was checked using an Olympus CX41 inverted light microscope (Olympus, Tokyo, Japan) equipped with an Olympus SP-500 UZ digital camera for 1, 3 and 7 days. 

### 2.7. Evaluation of Cytotoxicity of the Electrospun Membranes

The cytotoxic profile of the PCL/PLA and GelMA/ChMA nanofibrous membranes was evaluated using an MTT assay that was performed according to the guidelines set by ISO10933-5. Briefly, NHDF (10 × 10^3^ cells/well) were seeded in the presence of each fibrous mesh (n = 5), in 96-well plates, occupying < 10% of the well area, and then were incubated at 37 °C, in a 5% CO_2_ humidified atmosphere. After the incubation period (1, 3 and 7 days), the culture medium was removed and 50 µL of MTT (5 mg/mL in PBS) were added to each sample (n = 5), followed by their incubation for 4 h, at 37 °C, in a 5% CO_2_ atmosphere. After that, cells were treated with 200 µL of DMSO (0.04 N) for 30 min. Then, a microplate reader (Biorad xMark microplate spectrophotometer, Bio-Rad Laboratories, Hercules, CA, USA) was used to read the absorbance at 570 nm of the samples from each well. Wells containing cells in the culture medium without materials were used as negative control (K^−^), whereas cells cultured with EtOH 96% were used as a positive control (K^+^) [9,10,11]. Statistical analysis of the results was performed using one-way ANOVA with the Newman-Keuls post hoc test. 

### 2.8. Evaluation of Cell Adhesion at Surface of the Membranes

SEM analysis was done to evaluate the cell adhesion at membranes’ surface. To accomplish that, NHDF (2 × 10^4^ cells/well) were seeded at surface of the protective (PCL/PLA) as well as on the underlying (GelMA/ChMA) layer. After incubation period (1, 3 and 7 days), samples were fixed for 4 h with 2.5% (*v*/*v*) glutaraldehyde. Following this, samples were washed with PBS and dehydrated with growing concentrations of EtOH and then freeze-dried for 3 h. Finally, the samples were mounted on aluminum stubs with Araldite glue and sputter-coated with gold using a Quorum Q150R ES sputter coater (Quorum Technologies Ltd, Laughton, East Sussex, UK). SEM images were then captured with different magnifications, at an acceleration voltage of 20 kV, using a Hitachi S-3400N Scanning Electron Microscope (Hitachi, Tokyo, Japan) [10]. 

## 3. Results and Discussion

### 3.1. Nuclear Magnetic Resonance (NMR)

**Figure 1:**
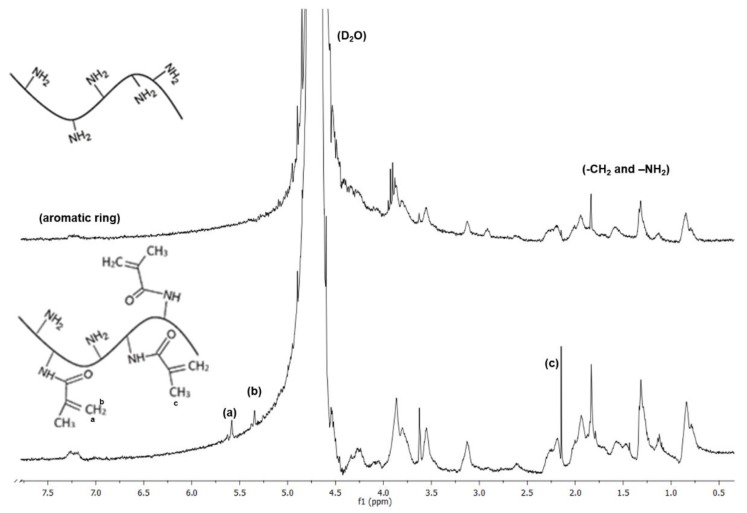
NMR spectra of the original and functionalized gelatin (GelMA).

NMR analysis was performed to confirm the success of the modification reaction of gelatin and chitosan with methacrylic anhydride. The gelatin spectra (Figure 1) shows the proton signals well resolved within the region of chemical shifts ranging from 0.7 to 3.8 ppm. 

**Figure 2:**
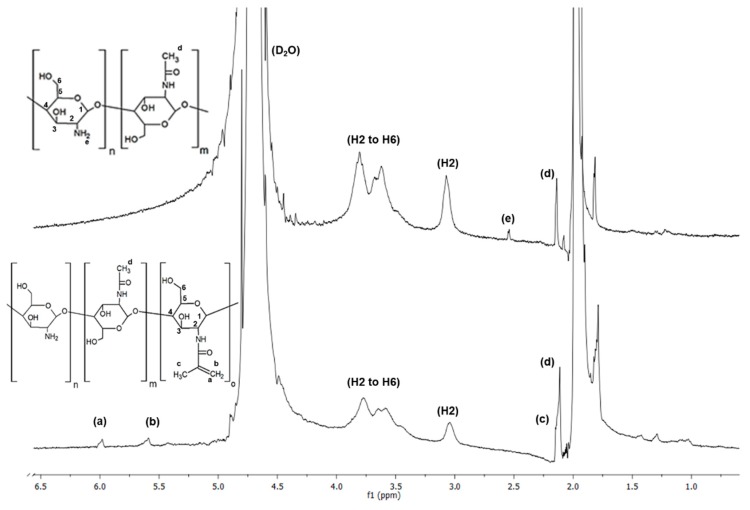
NMR spectra of the original and functionalized chitosan (ChMA).

These peaks could be assigned to the methyl resonances of specific amino acids and paired protons of –CH_2_– and –NH_2_ groups present in the molecular structure of gelatin [30,31]. A relatively weaker signal was observed at 7.2 ppm, which was assigned to the presence of aromatic ring [31]. The spectra of chitosan shown in Figure 2 confirmed the hydrogen peaks bonded to the third, fourth, fifth, and sixth carbon atoms of the glucosamine unit between 3.5 and 4 ppm. The hydrogen peak bonded to the second carbon of the glucosamine unit appeared at around 3.1 ppm and the methyl hydrogen peak of acetamido group at 2.6 ppm (e) [32,33]. 

By comparing the spectra of gelatin with GelMA and chitosan with ChMA, new peaks were observed in both GelMA (5.4 and 5.6 ppm) and ChMA (at 5.6 and 6 ppm), corresponding to the two protons of methacrylate double bonds (a) and (b), respectively. Furthermore, in GelMA spectrum (Figure 1), a peak at 2.15 ppm (c) was present, which was attributed to the hydrogens of the methacrylate methyl group. In ChMA spectrum, a peak at 1.9 ppm (c) was observed related to the methyl hydrogens of the acetyl and methacrylate groups. This peak was already present in the unmodified chitosan spectrum, due to the acetyl groups present in the base polymer [34,35,36]. From these results, it can be confirmed that the grafting reaction of MA into gelatin and chitosan occurred.

### 3.2. Fourier Transform Infrared-Attenuated Total Reflectance Spectroscopy (FTIR-ATR)

**Figure 3:**
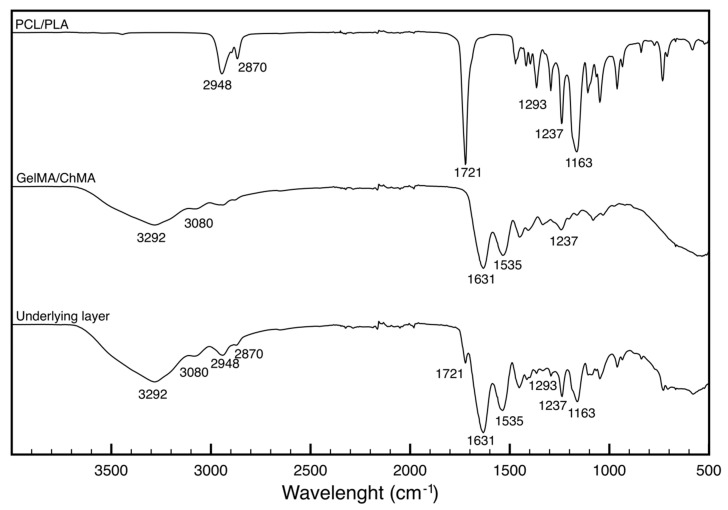
FTIR-ATR spectra of the individual PCL/PLA and GelMA/ChMA membranes and of the underlying layer of the membrane.

Figure 3 shows the FTIR-ATR spectra obtained for the PCL/PLA and GelMA/ChMA membranes as well as for the underlying layer of the PCL/PLA-GelMA/ChMA membrane, i.e., recorded from the GelMA/ChMA side.

PLA and PCL are aliphatic polyesters with similar structures, therefore PCL/PLA membrane spectrum presented the typical bands of the –CH_2_ asymmetrical stretching at 2948 cm^−1^ and the symmetrical stretching at 2870 cm^−1^. The characteristic stretching band of the carbonyl group (C = O) was present at 1721 cm^−1^ and the peak corresponding to the stretching of the C–C bonds at 1293 cm^−1^. Finally, at 1237 and 1163 cm^−1^, the peaks related to the stretching of the C–O–C bonds were visible (asymmetrical and symmetrical, respectively) [37].

The spectrum of GelMA/ChMA displayed the characteristic bands amide bands at 3292, 3080 and 1631 cm^−1^ assigned to the O–H, C–H and C=O stretches of the amide groups, respectively. The peaks at 1535 cm^−1^ was attributed to the N–H stretch of the amide (II) group [26].

The underlying side of the membrane spectra presented the characteristic peaks of each polymer. As expected, the bands assigned to the PCL were less evident than the ones assigned GelMA/ChMA since the latter was the membrane side that was analyzed. The characteristic ester C=O bond at the 1721, 2948 and 2870 cm^−1^ peaks related to the symmetrical and asymmetric stretching of the PCL –CH_2_ groups and the 1163 cm^−1^ band corresponding to the symmetrical stretching of the C–O–C bond of PCL/PLA, indicated the presence of PCL/PLA. On the other hand, the GelMA/ChMA presence was confirmed by amide I C=O stretching at 1631 cm^−1^, amide II in the region of 1535 cm^−1^ and amides A and B at 3292 and 3080 cm^−1^, respectively. Amide III was overlaid with the C–O–C asymmetric stretching band of PCL at 1237 cm^−1^. These results confirm the success of the deposition of the GelMA/ChMA layer on top of the PCL/PLA membrane.

### 3.3. Scanning Electronic Microscopy (SEM)

**Figure 4:**
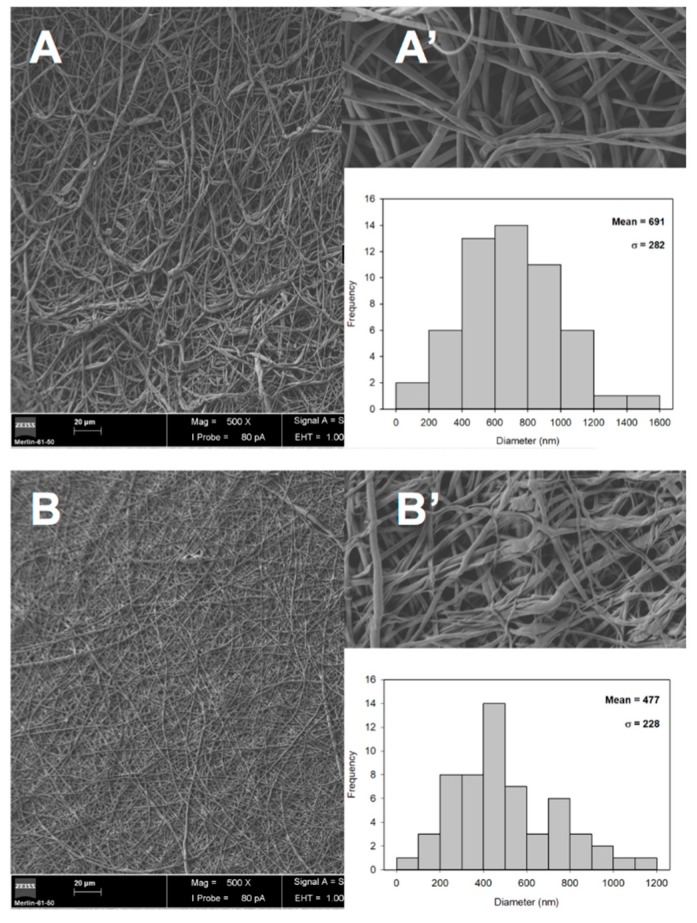
SEM images of the surface morphology of the PCL/PLA (**A**,**A’**) and GelMA/ChMA (**B**,**B’**) electrospun fibers Magnifications: 1000× (main figures) and 10,000× (inserted figures). The inserted graphics represent the individual measurements of the fiber diameters.

The morphology of the electrospun fibers of the protective and underlying layers of asymmetric membrane were observed by SEM, as shown in Figure 4. The obtained images show that both mixtures of PCL/PLA (Figure 4A,A’) and GelMA/ChMA (Figure 4B,B’) presented fibers with randomly orientation with smooth surfaces and without occurring the formation of beads. It is also shown in Figure 4A’ that some fibers composed of GelMA/ChMA present a ribbon-shaped morphology, which is very common in gelatin based electrospun fibers [38]. 

Moreover, the fibers diameter was determined through SEM images and the PCL/PLA membrane presented higher diameter values (691 ± 282 nm) than the GelMA/ChMA ones (477 ± 228 nm). Such result is in agreement with the data available in the literature, e.g. Casasola et al. produced nanofibers of PLA and with a fiber diameter around 685 ± 206 nm [39], while Dhandayuthapani et al. electrospun chitosan/gelatin nanofibers presenting a diameter ranging from 120–220 nm [40]. 

Moreover, it is noteworthy that the diameters of the produced membranes presented unimodal distribution and were within the range of collagen fibers (50–500 nm) found in natural ECM. Therefore, these results suggest that the produced membranes presented structural features that mimic the ECM of native skin, providing a suitable microenvironment for cell recruiting, adhesion, proliferation and ultimately improved skin tissue regeneration [41]. 

### 3.4. Dynamic Contact Angles Measurement

**Figure 5:**
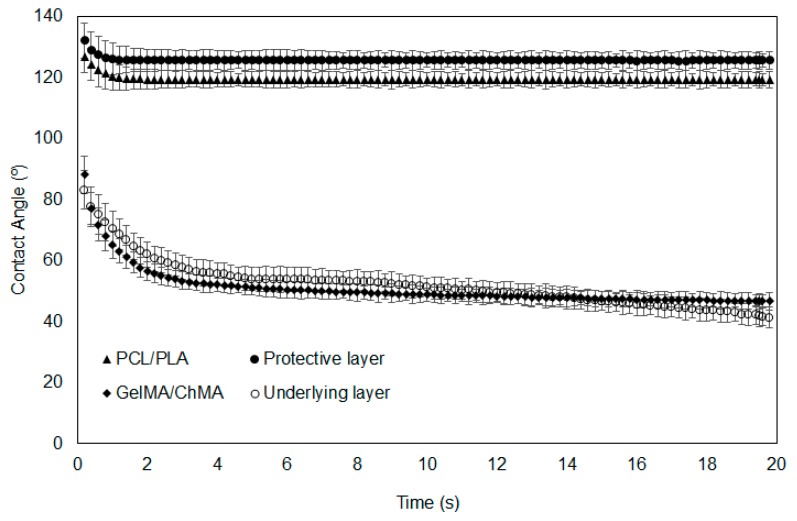
Determination of water contact angles at the surface of the nanofibrous membranes along time.

Water contact angles (WCA) were measured on the surface of the fibrous meshes prepared individually, as well on each layer of the asymmetric membranes, along time to assess their hydrophilicity/wettability (Figure 5). Surface wettability is considered a crucial parameter that influences cell adhesion, proliferation and differentiation. In the literature, it is described that cell adhesion is more favorable on moderate hydrophilic substrates (40° < WCA < 70°) than on hydrophobic (WCA > 90°) or very hydrophilic ones (WCA < 20°) [42].

In this study, PCL/PLA mesh exhibited an extremely high WCA (126.8° ± 5.7°), revealing its strong hydrophobic character and very poor wettability. The value of the WCA slightly decreased to a medium value of 125° remaining constant during the recorded time, which was also observed for the protective layer. Considering this similarity in the results, it is possible to perceive that the deposition of the underlying layer over the PCL/PLA one did not compromise its integrity and homogeneity. Thus, the PCL/PLA membranes presented a hydrophobic character due to the presence of the aliphatic polyester PCL and the thermoplastic polyester PLA [10,43,44]. This protective barrier wettability is desirable to avoid the external water infiltration, when applied as a protective layer in wound dressings [45]. 

Contrariwise, the WCA of the GelMA/ChMA fibers was initially 88.2° ± 6.1° and decreased gradually to 46.7° ± 2.2° after 20 s, revealing their good wettability. The same tendency was observed for the underlying layer when deposited over the PCL/PLA layer, meaning that the fabrication process did not interfere with the formation of the fibers and their uniform deposition. Such WCA values could be explained by the presence of hydrophilic groups (amine, carboxyl and hydroxyl groups) on chitosan and gelatin backbone, attributing the hydrophilic character of these membranes [19,26]. This property of underlying layer would allow absorbing wound exudates while maintaining a moist environment at wound site, which improves the healing process [46].

### 3.5. Weight Loss Evaluation

The majority of the commercially available wound dressings are non-degradable and, consequently, must be removed from the wound site, causing pain to the patient and possibly inducing the formation of scar tissues [47]. To avoid these situations, researchers have been developing biodegradable biomaterials that act as a temporary ECM. Ideally, the degradation rate of a polymeric matrices should match the rate of tissue formation to avoid any interference in tissue remodeling [48,49].

In the present work, this rate of degradation was controlled through the chemical crosslinking of the underlying layer. For this purpose, chitosan and gelatin were previously functionalized with methacrylic groups, sensible to undergo free radical polymerization. In this way, the crosslinking of these chemical groups occurred, when the membranes were exposed to UV light. 

To indirectly assess the efficiency of the photocrosslinking process, non-photocrosslinked (control) and photocrosslinked (UV irradiation) fibrous meshes prepared individually were incubated in PBS at 37 °C for three days, and their weight loss was determined. Simultaneously, PCL/PLA meshes were also submitted to the same process and their weight variation was also assessed. The obtained results are presented in Table 1.

As can be seen, the non-photocrosslinked GelMA/ChMA meshes lost 100% of their weight. After UV irradiation, this value significantly decreased to 5.63% ± 0.21%, demonstrating that the chemical photocrosslinking of both copolymers was successfully achieved. On the other hand, the weight loss values for PCL/PLA meshes were considerably lower, in both tested conditions. This fact was related with the hydrophobic nature of these polymers as well as their slow degradation profile [50].

### 3.6. Blood Compatibility

It is essential to evaluate the hemocompatibility of the membranes, since they are intended to interact with different compounds found at wound site, e.g. skin and blood cells. Blood compatibility has been widely studied to establish a standard for the possible interactions between the materials and blood [51]. In this way, herein, the hemolytic index, or percentage of hemolysis that each material causes in the red blood cells, and the thrombogenic capacity of the membranes were studied. 

#### 3.6.1. Hemolysis

**Figure 6:**
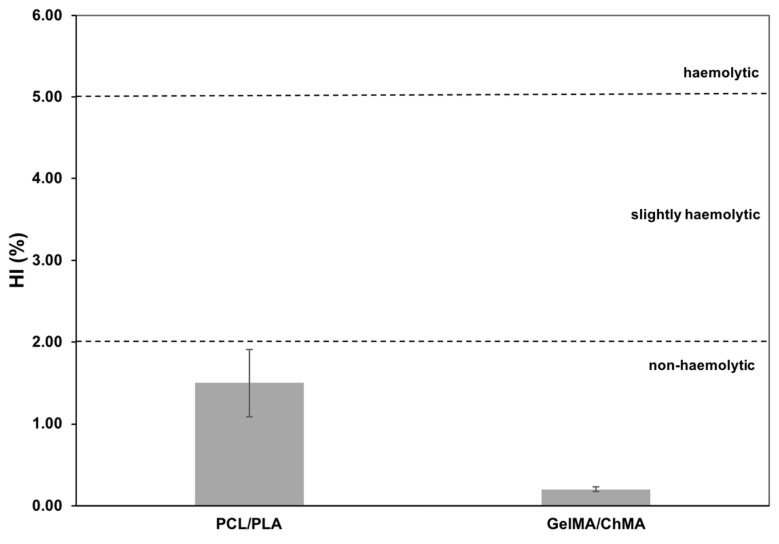
Determination of hemolysis index (HI) of ACD blood when placed in contact with the produced membranes. Data are expressed as mean ± SME (n = 6).

The hemolysis index when blood was in direct contact with both sides of the fibrous meshes showed to be non-hemolytic according to standard ASTM F 756-00, with a hemolysis index lower than 2%. The obtained results are presented in Figure 6 and are in line with the works presented by several authors, who stated that all mesh components are non-hemolytic [52,53,54], particularly the GelMA/ChMA meshes with an hemolytic index of 0.2% ± 0.03%, meaning that no significant disruption of the erythrocyte membranes occurred.

#### 3.6.2. Thrombogenicity

**Figure 7:**
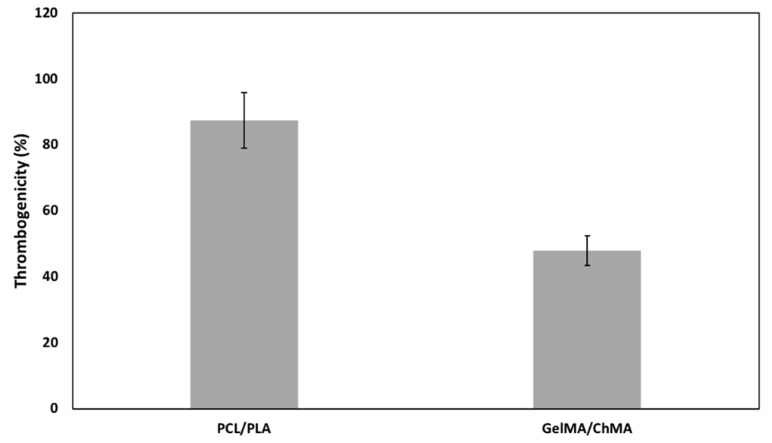
Percentage of thrombogenicity determined for each side of the asymmetric membrane after 40 min of contact with blood. Thrombogenicity was calculated and expressed as mean ± SME (n = 6).

The obtained structures were aimed to be used as wound dressing for skin regeneration. Thus, their surface induces a certain degree of thrombosis, which would improve the coagulation and consequently the wound healing process. The thrombus formation on the surface of each side of the asymmetric membrane was calculated after 40 min in direct contact with blood as the percentage of thrombogenicity. The results are shown in Figure 7. The PCL/PLA and GelMA/ChMA meshes presented 87.4% ± 8.5% and 47.9% ± 4.6%, respectively. These results can be explained by the hydrophilicity of the surfaces, since hydrophobic surfaces promote proteins adsorption followed by platelets adhesion and consequently to formation of the thrombus [52]. GelMA/ChMA meshes presented a more hydrophilic surface which may prevent the adsorption of proteins present in blood and inhibit some thrombus formation, leading to a lower, yet significant percentage of thrombogenicity. 

In summary, the blood compatibility results obtained for the asymmetric membranes reveal that they were not hemolytic but induce some thrombosis, making them good candidates for skin regeneration.

### 3.7. Evaluation of Proliferation of Fibroblasts in Presence of Nanofibrous Membranes

**Figure 8:**
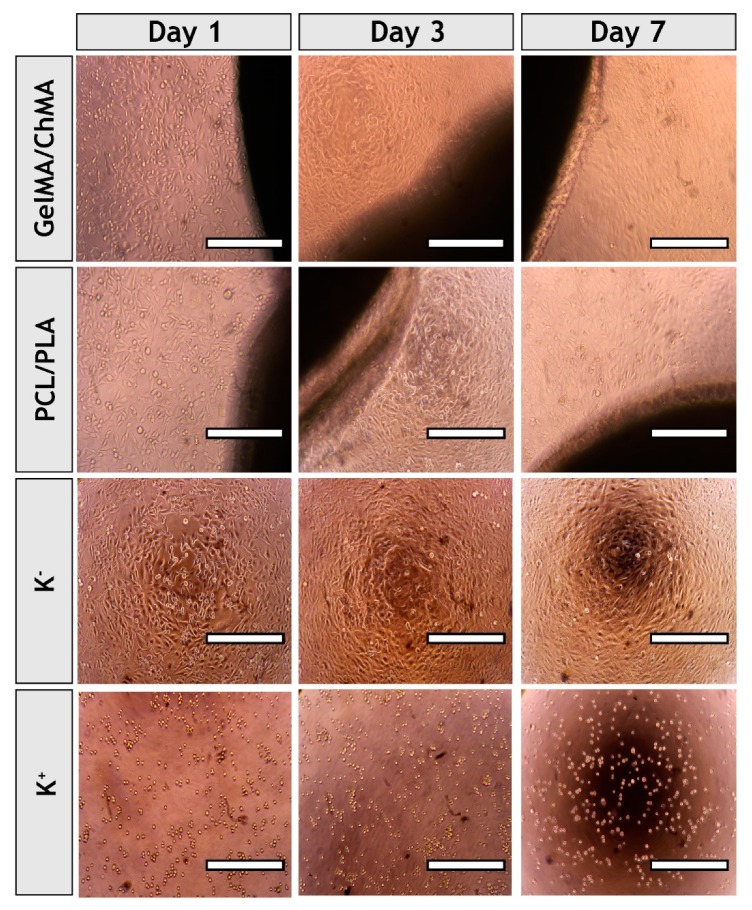
Optical microscopic photographs of NHDF cells in the presence of the each of the produced fibrous meshes after one, three and seven days of incubation. K^−^, negative control; K^+^, positive control. Original magnification, 100×.

It is known that wound healing is a complex and coordinated process [55]. Fibroblast cells have a critical role in this process, since these cells are involved in key events such as breaking down the fibrin clot, producing the new ECM and collagen structures and contracting the wound [56]. Thus, the cytocompatibility of each fibrous mesh was evaluated in contact with NHDF cells. The optical microscopic images of NHDF cells in contact with those meshes after one, three and seven days were acquired (see Figure 8). The images show that cells did not suffer any morphologic variation and cell spreading and elongation was visualized in groups treated with membranes and in the negative control (K^−^). Cells present in positive control (K^+^) displayed a spherical shape that is characteristic of dead cells.

### 3.8. Characterization of the Cytotoxicity Profile of the Membranes

**Figure 9:**
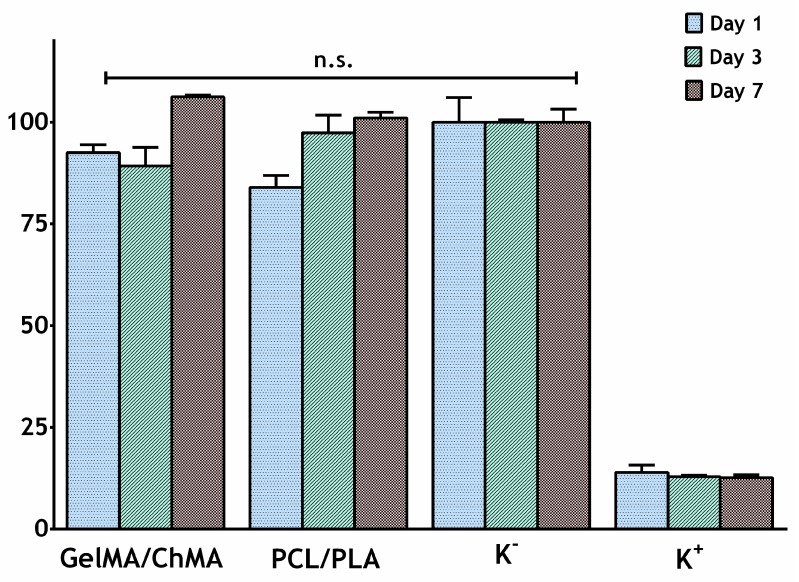
Evaluation of the NHDF cells viability after they be seeded in contact with nanofibrous membranes (PCL/PLA and ChMA/GelMA) for one, three and seven days. K^–^, negative control; K^+^, positive control. The statistical analysis of the results was performed using one-way ANOVA with Newman-Keuls test. Result are expressed as mean ± SD (n = 3).

In addition, the materials’ cytocompatibility was also characterized through an MTT assay, for one, three and seven days. This assay is based on the metabolic conversion of the MTT, where a yellow tetrazole salt is converted to purple formazan crystals in living cells. Such process is proportional to the number of viable cells present in each well [57]. The results presented in Figure 9 show that the fibroblasts cells viability was not affected after seven days of being in contact with the protective and underlying layers individually, since there was no statistical difference between layer groups and negative control (where cells were incubated only cell culture medium). Furthermore, the degradation byproducts be produced during seven days of the assay did not impair the cell activity, which is also vital to support the application of these membranes for the aimed biomedical application. 

### 3.9. Evaluation of Cell Adhesion at Surface of the Membranes

**Figure 10:**
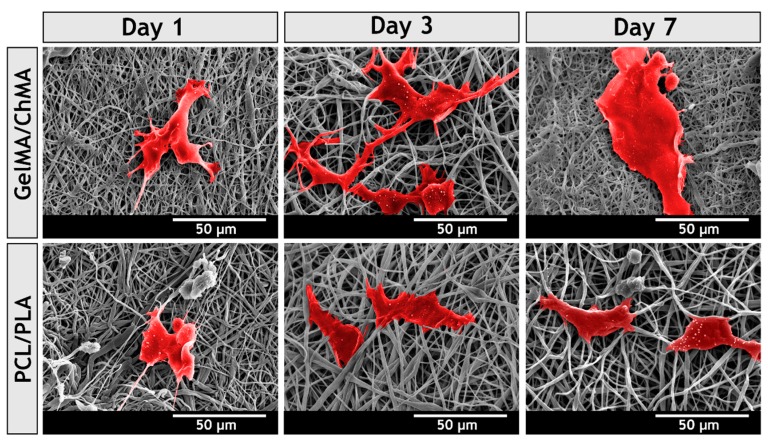
SEM micrographs of NHDF cells morphology adhered on the surface of the different produced membranes, after 1, 3 and 7 days.

To further characterize the interaction between NHDF cells and nanofibers, SEM analysis was performed. Figure 10 shows that both layers provided a 3D structure for cellular adhesion, growth and proliferation. However, the attachment and spreading of fibroblasts was improved in underlying layer, which was produced with chitosan and gelatin. After three days, the cells already presented the typical fibroblastic morphology, lamellipodia connecting to surrounding mesh were visualized and a continuous layer of cells was observed after seven days. This is in accordance with results previously reported by Zhang et al., who showed cells become more spread on the surface of PCL/gelatin membranes than on PCL ones [58]. 

The cell behavior on the surface of the underlying layer can be explained by the Arg-Gly-Asp (RGD)-like sequences of gelatin, which is known as the most effective peptides sequence for stimulating cell adhesion on materials surface [59,60]. The integrin receptors available on cell surface recognize these specific sequences, allowing cells to anchor as well as signal transduction mechanisms [61,62]. Such process is usually comprised of four different partly overlapping events: cell attachment, cell spreading, organization of actin cytoskeleton and formation of focal adhesions [59]. All these events are fundamental for tissue regeneration. 

The ameliorated bioadhesive properties of the underlying layer demonstrated their potential to improve the wound healing process by promoting cell adhesion and proliferation. Once fibroblasts are at wound site, they produce and secrete ECM proteins, and growth factors that are required for the re-establishment of the structure of the injured tissue. 

## 4. Conclusions

In this work asymmetric membranes were usefully prepared through the electrospinning technique. These membranes demonstrated a great potential to be used in the wound healing process since they presented an upper layer able to protect the wound from the environment and a hydrophilic underlying layer capable of providing a moist environment at the wound site. Moreover, in vitro assays demonstrated that the membranes were hemocompatible, inducing some thrombosis essential to control the hemostasis phase of healing process. Further, fibroblast cells were able to adhere, spread and proliferate at their surface. Additionally, these membranes were biodegradable, allowing to tailor the degradation rate of polymeric matrices to match the rate of tissue formation, which avoids the removal/substitution of the wound dressing. 

## Figures and Tables

**Table 1:** Assessed values of weight loss (%) for both the protective (PCL/PLA) and underlying layer (GelMA/ChMA) of the asymmetrical membranes.

Sample	Weight Loss (%)	
	Control	UV Irradiation
PCL/PLA	1.23 ± 0.34	1.54 ± 0.22
GelMA/ChMA	100.00 ± 0.00	5.63 ± 0.21
